# Physiological changes in pregnancy

**DOI:** 10.5830/CVJA-2016-021

**Published:** 2016

**Authors:** Priya Soma-Pillay, Nelson-Piercy Catherine, Heli Tolppanen, Alexandre Mebazaa, Heli Tolppanen, Alexandre Mebazaa

**Affiliations:** Department of Obstetrics and Gynaecology, University of Pretoria and Steve Biko Academic Hospital, Pretoria, South Africa; Department of Obstetric Medicine, Women’s Health Academic Centre, King’s Health Partners; Guy’s and St Thomas’ Foundation Trust, and Queen Charlotte’s and Chelsea Hospital, Imperial College Healthcare Trust, London, UK; INSERM UMRS 942, Paris, France; INSERM UMRS 942, Paris, France; Heart and Lung Centre, Helsinki University Central Hospital, Finland; University Paris Diderot, Sorbonne Paris Cité, Paris; Department of Anesthesia and Critical Care, Hôpital Lariboisière, APHP, France

**Keywords:** hypercoagulable state, diabetogenic, uterine contractions

## Abstract

Physiological changes occur in pregnancy to nurture the developing foetus and prepare the mother for labour and delivery. Some of these changes influence normal biochemical values while others may mimic symptoms of medical disease. It is important to differentiate between normal physiological changes and disease pathology. This review highlights the important changes that take place during normal pregnancy.

## Abstract

During pregnancy, the pregnant mother undergoes significant anatomical and physiological changes in order to nurture and accommodate the developing foetus. These changes begin after conception and affect every organ system in the body.^1^ For most women experiencing an uncomplicated pregnancy, these changes resolve after pregnancy with minimal residual effects. It is important to understand the normal physiological changes occurring in pregnancy as this will help differentiate from adaptations that are abnormal.

## 

Plasma volume increases progressively throughout normal pregnancy.^2^ Most of this 50% increase occurs by 34 weeks’ gestation and is proportional to the birthweight of the baby. Because the expansion in plasma volume is greater than the increase in red blood cell mass, there is a fall in haemoglobin concentration, haematocrit and red blood cell count. Despite this haemodilution, there is usually no change in mean corpuscular volume (MCV) or mean corpuscular haemoglobin concentration (MCHC).

The platelet count tends to fall progressively during normal pregnancy, although it usually remains within normal limits. In a proportion of women (5–10%), the count will reach levels of 100–150 × 10^9^ cells/l by term and this occurs in the absence of any pathological process. In practice, therefore, a woman is not considered to be thrombocytopenic in pregnancy until the platelet count is less than 100 × 10^9^ cells/l.

Pregnancy causes a two- to three-fold increase in the requirement for iron, not only for haemoglobin synthesis but also for for the foetus and the production of certain enzymes. There is a 10- to 20-fold increase in folate requirements and a two-fold increase in the requirement for vitamin B_12_.

Changes in the coagulation system during pregnancy produce a physiological hypercoagulable state (in preparation for haemostasis following delivery).^3^ The concentrations of certain clotting factors, particularly VIII, IX and X, are increased. Fibrinogen levels rise significantly by up to 50% and fibrinolytic activity is decreased. Concentrations of endogenous anticoagulants such as antithrombin and protein S decrease. Thus pregnancy alters the balance within the coagulation system in favour of clotting, predisposing the pregnant and postpartum woman to venous thrombosis. This increased risk is present from the first trimester and for at least 12 weeks following delivery. *In vitro* tests of coagulation [activated partial thromboplastin time (APTT), prothrombin time (PT) and thrombin time (TT)] remain normal in the absence of anticoagulants or a coagulopathy.

Venous stasis in the lower limbs is associated with venodilation and decreased flow, which is more marked on the left. This is due to compression of the left iliac vein by the left iliac artery and the ovarian artery. On the right, the iliac artery does not cross the vein.

## Cardiac changes

Changes in the cardiovascular system in pregnancy are profound and begin early in pregnancy, such that by eight weeks’ gestation, the cardiac output has already increased by 20%. The primary event is probably peripheral vasodilatation. This is mediated by endothelium-dependent factors, including nitric oxide synthesis, upregulated by oestradiol and possibly vasodilatory prostaglandins (PGI2). Peripheral vasodilation leads to a 25–30% fall in systemic vascular resistance, and to compensate for this, cardiac output increases by around 40% during pregnancy. This is achieved predominantly via an increase in stroke volume, but also to a lesser extent, an increase in heart rate. The maximum cardiac output is found at about 20–28 weeks’ gestation. There is a minimal fall at term.

An increase in stroke volume is possible due to the early increase in ventricular wall muscle mass and end-diastolic volume (but not end-diastolic pressure) seen in pregnancy. The heart is physiologically dilated and myocardial contractility is increased. Although stroke volume declines towards term, the increase in maternal heart rate (10–20 bpm) is maintained, thus preserving the increased cardiac output. Blood pressure decreases in the first and second trimesters but increases to non-pregnant levels in the third trimester

There is a profound effect of maternal position towards term upon the haemodynamic profile of both the mother and foetus. In the supine position, pressure of the gravid uterus on the inferior vena cava (IVC) causes a reduction in venous return to the heart and a consequent fall in stroke volume and cardiac output. Turning from the lateral to the supine position may result in a 25% reduction in cardiac output. Pregnant women should therefore be nursed in the left or right lateral position wherever possible. If the woman has to be kept on her back, the pelvis should be rotated so that the uterus drops to the side and off the IVC, and cardiac output and uteroplacental blood flow are optimised. Reduced cardiac output is associated with a reduction in uterine blood flow and therefore in placental perfusion, which could compromise the foetus.

Although both blood volume and stroke volume increase in pregnancy, pulmonary capillary wedge pressure and central venous pressure do not increase significantly. Pulmonary vascular resistance (PVR), like systemic vascular resistance (SVR), decreases significantly in normal pregnancy. Although there is no increase in pulmonary capillary wedge pressure (PCWP), serum colloid osmotic pressure is reduced by 10–15%. The colloid osmotic pressure/pulmonary capillary wedge pressure gradient is reduced by about 30%, making pregnant women particularly susceptible to pulmonary oedema. Pulmonary oedema will be precipitated if there is either an increase in cardiac pre-load (such as infusion of fluids) or increased pulmonary capillary permeability (such as in pre-eclampsia) or both.

Labour is associated with further increases in cardiac output (15% in the first stage and 50% in the second stage) Uterine contractions lead to an auto-transfusion of 300–500 ml of blood back into the circulation and the sympathetic response to pain and anxiety further elevate the heart rate and blood pressure. Cardiac output is increased between contractions but more so during contractions.

Following delivery there is an immediate rise in cardiac output due to relief of the inferior vena cava obstruction and contraction of the uterus, which empties blood into the systemic circulation. Cardiac output increases by 60–80%, followed by a rapid decline to pre-labour values within about one hour of delivery. Transfer of fluid from the extravascular space increases venous return and stroke volume further.

Those women with cardiovascular compromise are therefore most at risk of pulmorary oedema during the second stage of labour and the immediate postpartum period. Cardiac output has nearly returned to normal (pre-pregnancy values) two weeks after delivery, although some pathological changes (e.g. hypertension in pre-eclampsia) may take much longer.

The above physiological changes lead to changes on cardiovascular examination that may be misinterpreted as pathological by those unfamiliar with pregnancy. Changes may include a bounding or collapsing pulse and an ejection systolic murmur, present in over 90% of pregnant women. The murmur may be loud and audible all over the precordium, with the first heart sound loud and possibly sometimes a third heart sound. There may be ectopic beats and peripheral oedema.

Normal findings on ECG in pregnancy that may partly relate to changes in the position of the heart include:

atrial and ventricular ectopics
Q wave (small) and inverted T wave in lead III
ST-segment depression and T-wave inversion in the inferior and lateral leads
left-axis shift of QRS.

## Adaptive changes in renal vasculature

The primary adaptive mechanism in pregnancy is a marked fall in systemic vascular resistance (SVR) occurring by week six of gestation. The 40% fall in SVR also affects the renal vasculature.^4^ Despite a major increase in plasma volume during pregnancy, the massive decrease in SVR creates a state of arterial under-filling because 85% of the volume resides in the venous circulation.^5^ This arterial under-filling state is unique to pregnancy. The fall in SVR is combined with increased renal blood flow and this is in contrast to other states of arterial under-filling, such as cirrhosis, sepsis or arterio-venous fistulas.^3^,^6^

Relaxin, a peptide hormone produced by the corpus luteum, decidua and placenta, plays an important role in the regulation of haemodynamic and water metabolism during pregnancy. Serum concentrations of relaxin, already elevated in the luteal phase of the menstrual cycle, rise after conception to a peak at the end of the first trimester and fall to an intermediate value throughout the second and third trimester. Relaxin stimulates the formation of endothelin, which in turn mediates vasodilation of renal arteries via nitric oxide (NO) synthesis.^7^

Despite activation of the renin–angiotensin–aldosterone (RAA) system in early pregnancy, a simultaneous relative resistance to angiotensin II develops, counterbalancing the vasoconstrictive effect and allowing profound vasodilatation.^8^ This insensitivity to angiotensin II may be explained by the effects of progesterone and vascular endothelial growth factormediated prostacyclin production, as well as modifications in the angiotensin I receptors during pregnancy.^9^ The vascular refractoriness to angiotensin II may also be shared by other vasoconstrictors such as adrenergic agonists and arginine vasopressin (AVP).^10^ It is possible that in the second half of pregnancy, the placental vasodilatators are more important in the maintenance of the vasodilatatory state.^6^

## Changes in renal anatomy and function

As a consequence of renal vasodilatation, renal plasma flow and glomerular filtration rate (GFR) both increase, compared to non-pregnant levels, by 40–65 and 50–85%, respectively. In addition, the increase in plasma volume causes decreased oncotic pressure in the glomeruli, with a subsequent rise in GFR.^11^ Vascular resistance decreases in both the renal afferent and efferent arterioles and therefore, despite the massive increase in renal plasma flow, glomerular hydrostatic pressure remains stable, avoiding the development of glomerular hypertension. As the GFR rises, both serum creatinine and urea concentrations decrease to mean values of about 44.2 μmol/l and 3.2 mmol/l, respectively.

The increased renal blood flow leads to an increase in renal size of 1–1.5 cm, reaching the maximal size by mid-pregnancy. The kidney, pelvis and calyceal systems dilate due to mechanical compressive forces on the ureters. Progesterone, which reduces ureteral tone, peristalsis and contraction pressure, mediates these anatomical changes.^11^ The increase in renal size is associated with an increase in renal vasculature, interstitial volume and urinary dead space. There is also dilation of the ureters, renal pelvis and calyces, leading to physiological hydronephrosis in over 80% of women.^12^ There is often a right-sided predominance of hydronephrosis due to the anatomical circumstances of the right ureter crossing the iliac and ovarian vessels at an angle before entering the pelvis. Urinary stasis in the dilated collecting system predisposes pregnant women with asymptomatic bacteriuria to pyelonephritis.^12^

There are also alterations in the tubular handling of wastes and nutrients. As in the non-pregnant state, glucose is freely filtered in the glomerulus. During pregnancy, the reabsorption of glucose in the proximal and collecting tubule is less effective, with variable excretion. About 90% of pregnant women with normal blood glucose levels excrete 1–10 g of glucose per day. Due to the increases in both GFR and glomerular capillary permeability to albumin, the fractional excretion of protein may increase up to 300 mg/day and protein excretion also increases. In normal pregnancies the total protein concentration in urine does not increase above the upper normal limit. Uric acid excretion also increases due to increased GFR and/or decreased tubular reabsorption.^11^

## Body water metabolism

Arterial under-filling in pregnancy leads to the stimulation of arterial baroreceptors, activating the RAA and the sympathetic nervous systems. This results in a non-osmotic release of AVP from the hypothalamus. These changes lead to sodium and water retention in the kidneys and create a hypervolaemic, hypoosmolar state characteristic of pregnancy.^6^ Extracellular volume increases by 30–50% and plasma volume by 30–40%. Maternal blood volume increases by 45% to approximately 1 200 to 1 600 ml above non-pregnant values. By the late third trimester the plasma volume increases by more than 50–60%, with a lower increase in red blood cell mass, and therefore plasma osmolality falls by 10 mosmol/kg. The increase in plasma volume plays a critical role in maintaining circulating blood volume, blood pressure and uteroplacental perfusion during pregnancy.^13^

Activation of the RAA system leads to increased plasma levels of aldosterone and subsequent salt and water retention in the distal tubule and collecting duct. In addition to the increased renin production by the kidneys, ovaries and uteroplacental unit produce an inactive precursor protein of renin in early pregnancy.^14^ The placenta also produces oestrogens that stimulate the synthesis of angiotensinogen by the liver, resulting in proportionally increased levels of aldosterone compared to renin. Plasma levels of aldosterone correlate well with those of oestrogens and rise progressively during pregnancy. The increase in aldosterone is responsible for the increase in plasma volume during pregnancy.^13^ Progesterone, which is a potent aldosterone antagonist, allows natriuresis despite the sodium-retaining properties of aldosterone. The rise in GFR also increases distal sodium delivery, allowing excretion of excess sodium. Progesterone has antikaliuretic effects and therefore excretion of potassium is kept constant throughout pregnancy due to changes in tubular reabsorption, and total body potassium increases during pregnancy.^6^,^15^

Hypothalamic AVP release increases early in pregnancy as a result of increased relaxin levels. AVP mediates an increase in water reabsorption via aquaporin 2 channels in the collecting duct. The threshold for hypothalamic secretion of AVP and the threshold for thirst is reset to a lower plasma osmolality level, creating the hypo-osmolar state characteristic of pregnancy. These changes are mediated by human chorionic gonadotropin (hCG) and relaxin.^11^,^16^

In middle and late pregnancy there is a four-fold increase in vasopressinase, an aminopeptidase produced by the placenta. These changes enhance the metabolic clearance of vasopressin and regulate the levels of active AVP. In conditions of increased placental production of vasopressinase, such as pre-eclampsia or twin pregnancies, a transient diabetes insipidus may develop.^17^ As a consequence of this volume expansion, the secretion of atrial natriuretic peptides increases by 40% in the third trimester, and rises further during the first week postpartum. The levels of natriuretic peptides are higher in pregnant women with chronic hypertension and pre-eclampsia.^18^

## Respiratory changes

There is a significant increase in oxygen demand during normal pregnancy. This is due to a 15% increase in the metabolic rate and a 20% increased consumption of oxygen. There is a 40–50% increase in minute ventilation, mostly due to an increase in tidal volume, rather than in the respiratory rate. This maternal hyperventilation causes arterial pO2 to increase and arterial pCO_2_ to fall, with a compensatory fall in serum bicarbonate to 18–22 mmol/l (see Table 1). A mild fully compensated respiratory alkalosis is therefore normal in pregnancy (arterial pH 7.44).

**Table 1:** 1. Reference ranges for respiratory function in pregnancy

	*Normal values*	
*Investigations*	*Pregnant*	*Non-pregnant*
pH	7.40–7.47	7.35–7.45
pCO_2_, mmHg (kPa)	≤ 30 (3.6–4.3)	35–40 (4.7–6.0)
pO_2_, mmHg (kPa)	100–104 (12.6–14.0)	90–100 (10.6–14.0)
Base excess	No change	+2 to –2
Bicarbonate (mmol/l)	18–22	20–28

**Fig. 1.**
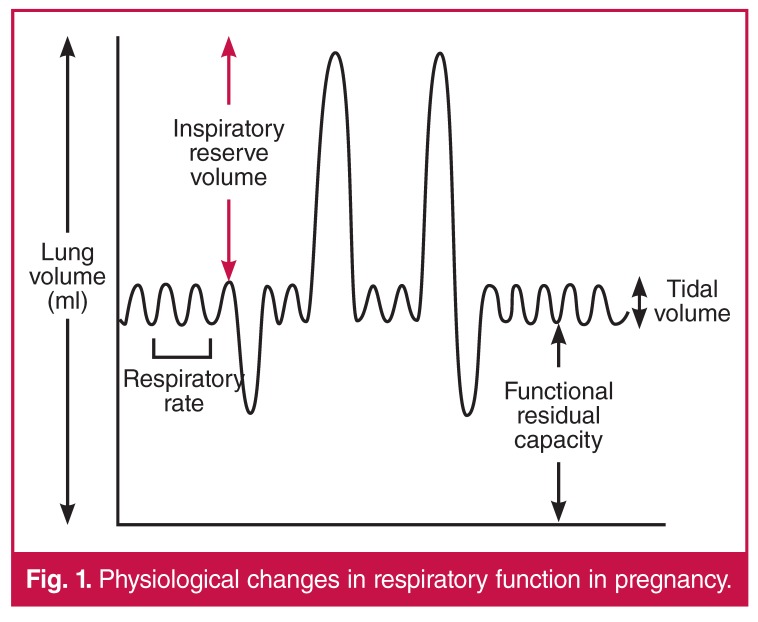
Physiological changes in respiratory function in pregnancy.

Diaphragmatic elevation in late pregnancy results in decreased functional residual capacity but diaphragmatic excursion and therefore vital capacity remain unaltered. Inspiratory reserve volume is reduced early in pregnancy, as a result of increased tidal volume, but increases in the third trimester, as a result of reduced functional residual capacity (see Fig. 1). Peak expiratory flow rate (PEFR) and forced expiratory volume in one second (FEV_1_) are unaffected by pregnancy.

Pregnancy may also be accompanied by a subjective feeling of breathlessness without hypoxia. This is physiological and is most common in the third trimester but may start at any time during gestation. Classically, the breathlessness is present at rest or while talking and may paradoxically improve during mild activity.

## Adaptive changes in the alimentary tract

Nausea and vomiting are very common complaints in pregnancy, affecting 50–90% of pregnancies.^19^ This might be an adaptive mechanism of pregnancy, aiming at preventing pregnant women from consuming potentially teratogenic substances such as strong-tasting fruits and vegetables. The exact underlying mechanism is not clear but pregnancy-associated hormones such as human chorionic gonadotropin (hCG), oestrogen and progesterone could to be involved in the aetiology. The levels of hCG peak at the end of the first trimester when the trophoblast is most actively producing hCG, correlating with the nausea symptoms. Nausea is also more frequent in pregnancies with high levels of hCG, such as in twin pregnancies.

Thyroid hormones may also be involved in the development of nausea symptoms, as a strong association with nausea and abnormal thyroid function tests has been found. Thyroid-stimulating hormone (TSH) and hCG have similar biomolecular structures and therefore hCG cross-reacts with TSH, stimulating the thyroid gland.^18^ Psychological causes, genetic incompatibility, immunological factors, nutritional deficiencies as well as *Helicobacter pylori* infection have been proposed as aetiological factors of nausea and vomiting during pregnancy.^20^

The nausea symptoms usually resolve by week 20 but about 10–20% of the patients experience symptoms beyond week 20 and some until the end of the pregnancy.^21^ In most cases minor dietary modification and observation of electrolyte balance is sufficient. About 0.5–3% of pregnant women develop hyperemesis gravidum, a severe form of nausea and excessive vomiting, often resulting in dehydration, electrolyte imbalance, ketonuria, weight loss and vitamin or mineral deficiencies.^19^,^21^ In these cases intravenous fluid and vitamin substitution is commonly required. Thiamine supplementation is important in order to avoid the development of Wernicke’s encephalopathy.^22^

As pregnancy progresses, mechanical changes in the alimentary tract also occur, caused by the growing uterus. The stomach is increasingly displaced upwards, leading to an altered axis and increased intra-gastric pressure. The oesophageal sphincter tone is also decreased and these factors may predispose to symptoms of reflux, as well as nausea and vomiting.^23^

Changes in oestrogen and progesterone levels also influence the structural alterations in the gastrointestinal tract. These include abnormalities in gastric neural activity and smooth muscle function, leading to gastic dysrhythmia or gastroparesis. The alterations are pronounced in women with pre-existing gastrointestinal diseases such as gastroesophageal reflux disease, diabetic gastroparesis, gastric bypass surgery or inflammatory bowel disease.^21^,^23^

## Endocrine changes

## Thyroid

There is an increase in the production of thyroxine-binding globulin (TBG) by the liver, resulting in increased levels of thyroxine (T_4_) and tri-iodothyronine (T_3_). Serum free T_4_ (fT_4_) and T_3_ (fT_3_) levels are slightly altered but are usually of no clinical significance. Levels of free T_3_ and T_4_ do however decrease slightly in the second and third trimesters of pregnancy and the normal ranges are reduced.^24^ Free T_3_ and T_4_ are the physiologically important hormones and are the main determinants of whether a patient is euthyroid.

Serum concentrations of TSH are decreased slightly in the first trimester in response to the thyrotropic effects of increased levels of human chorionic gonadotropin. Levels of TSH increase again at the end of the first trimester, and the upper limit in pregnancy is raised to 5.5 μmol/l compared with the level of 4.0 μmol/l in the non-pregnant state (Table 2).

**Table 2:** Reference ranges for thyroid function in pregnancy^37^

*Thyroid function*	*Non-pregnant*	*1st trimester*	*2nd trimester*	*3rd trimester*
fT_4_ (pmol/l)	9–26	10–16	9–15.5	8–14.5
fT_3_ (pmol/l)	2.6–5.7	3–7	3–5.5	2.5–5.5
TSH (mU/l)	0.3–4.2	0–5.5	0.5–3.5	0.5–3.5

Pregnancy is associated with a relative iodine deficiency. The causes for this are active transport of iodine from the mother to the foeto-placental unit and increased iodine excretion in the urine. The World Health Organisation recommends an increase in iodine intake in pregnancy from 100 to 150–200 mg/day.^24^ If iodine intake is maintained in pregnancy, the size of the thyroid gland remains unchanged and therefore the presence of goiter should always be investigated. The thyroid gland is 25% larger in patients who are iodine deficient.

## Adrenal gland

Three types of steroids are produced by the adrenal glands: mineralocorticoids, glucocorticoids and sex steroids. The RAA system is stimulated due to reductions in vascular resistance and blood pressure, causing a three-fold increase in aldosterone levels in the first trimester and a 10-fold increase in the third trimester.^25^,^26^ Levels of angiotensin II are increased two- to four-fold and renin activity is increased three to four times that of non-pregnant values.

During pregnancy there is also an increase in serum levels of deoxycorticosterone, corticosteroid-binding globulin (CBG), adrenocorticotropic hormone (ACTH), cortisol and free cortisol. These changes cause a state of physiological hypercortisolism and may be clinically manifested by the striae, facial plethora, rising blood pressure or impaired glucose tolerance.^27^ Total cortisol levels increase at the end of the first trimester and are three times higher than non-pregnant values at the end of pregnancy. Hypercortisolism in late pregnancy is also the result of the production of corticotropin-releasing hormone by the placenta – one of the triggers for the onset of labour. Diurnal variations in ACTH and cortisol levels are maintained. The hypothalamic–pituitary axis response to exogenous glucocorticoids is blunted during pregnancy.

## Pituitary gland

The pituitary gland enlarges in pregnancy and this is mainly due to proliferation of prolactin-producing cells in the anterior lobe. Serum prolactin levels increase in the first trimester and are 10 times higher at term. The increase in prolactin is most likely due to increasing serum oestradiol concentrations during pregnancy. Levels of follicle-stimulating hormone (FSH) and luteinising hormone (LH) are undetectable during pregnancy due to the negative feedback from elevated levels of oestrogen, progesterone and inhibin.^28^ Pituitary growth hormone production is decreased but serum growth hormone levels are increased due to growth hormone production from the placenta.

The posterior pituitary produces oxytocin and arginine vasopressin (AVP). Oxytocin levels increase in pregnancy and peak at term. Levels of antidiuretic hormone (ADH) remain unchanged but the decrease in sodium concentration in pregnancy causes a decrease in osmolality. There is therefore a resetting of osmoreceptors for ADH release and thirst.^29^

## Glucose metabolism

Pregnancy is a diabetogenic state and the adaptations in glucose metabolism allow shunting of glucose to the foetus to promote development, while maintaining adequate maternal nutrition.^30^ Insulin-secreting pancreatic beta-cells undergo hyperplasia, resulting in increased insulin secretion and increased insulin sensitivity in early pregnancy, followed by progressive insulin resistance.^31^

Maternal insulin resistance begins in the second trimester and peaks in the third trimester. This is the result of increasing secretion of diabetogenic hormones such as human placental lactogen, growth hormone, progesterone, cortisol and prolactin. These hormones cause a decrease in insulin sensitivity in the peripheral tissues such as adipocytes and skeletal muscle by interfering with insulin receptor signalling.^32^ The effect of the placental hormones on insulin sensitivity is made evident postpartum when there is a sudden decrease in insulin resistance.^33^

Insulin levels are increased in both the fasting and postprandial states in pregnancy. Fasting glucose levels are however decreased due to:

increased storage of tissue glycogen
increased peripheral glucose use
decrease in glucose production by the liver
uptake of glucose by the foetus.^34^

Insulin resistance and relative hypoglycaemia results in lipolysis, allowing the pregnant mother to preferentially use fat for fuel, preserving the available glucose and amino acids for the foetus and minimising protein catabolism. The placenta allows transfer of glucose, amino acids and ketones to the foetus but is impermeable to large lipids. If a woman’s endocrine pancreatic function is impaired, and she is unable to overcome the insulin resistance associated with pregnancy then gestational diabetes develops.

## Lipid metabolism

There is an increase in total serum cholesterol and triglyceride levels in pregnancy. The increase in triglyceride levels is mainly as a result of increased synthesis by the liver and decreased lipoprotein lipase activity, resulting in decreased catabolism of adipose tissue. Low-density lipoprotein (LDL) cholesterol levels also increase and reach 50% at term. High-density lipoprotein levels increase in the first half of pregnancy and fall in the third trimester but concentrations are 15% higher than non-pregnant levels.

Changes in lipid metabolism accommodate the needs of the developing foetus. Increased triglyceride levels provide for the mother’s energy needs while glucose is spared for the foetus. The increase in LDL cholesterol is important for placental steroidogenesis.

## Protein metabolism

Pregnant women require an increased intake of protein during pregnancy. Amino acids are actively transported across the placenta to fulfill the needs of the developing foetus. During pregnancy, protein catabolism is decreased as fat stores are used to provide for energy metabolism.

## Calcium metabolism

The average foetus requires about 30 g of calcium to maintain its physiological processes. Most of this calcium is transferred to the foetus during the third trimester and is derived from increased dietary absorption by the mother.^35^ There is a decrease in total serum calcium concentration during pregnancy. This is mainly due to a decrease in serum albumin levels due to haemodilution, resulting in a decrease in the albumin-bound fraction of calcium. However the physiologically important fraction, serum ionised calcium, remains unchanged.^36^ Therefore maternal serum levels of calcium are maintained during pregnancy and foetal needs are met by increased intestinal absorption, which doubles from 12 weeks’ gestation. However the peak demand for calcium is only in the third trimester. This early increase in calcium absorption may allow the maternal skeleton to store calcium in advance.^17^

Serum levels of 25-hydroxyvitamin D increase and this is metabolised further into 1.25-dihydroxyvitamin D. The increase in 1.25-dihydroxyvitamin D is directly responsible for the increase in intestinal calcium absorption.^36^

Increased calcium absorption is associated with an increase in calcium excretion in the urine and these changes begin from 12 weeks. During periods of fasting, urinary calcium values are low or normal, confirming that hypercalciuria is the consequence of increased absorption.^35^ Pregnancy is therefore a risk factor for kidney stones.

## Skeletal and bone density changes

There is controversy regarding the effect of pregnancy on maternal bone loss. Although pregnancy and lactation are associated with reversible bone loss, studies do not support an association between parity and osteoporosis in later life.^25^ Bone turnover is low in the first trimester and increases in the third trimester when foetal calcium needs are increased. The source of the calcium in the third trimester is previously stored skeletal calcium.^36^

A study of bone biopsies in pregnancy has shown a change in the micro-architectural pattern of bone in pregnancy but not overall bone mass.^36^ The changes reflect the need for the maternal skeleton to be resistant to bending forces and biochemical stresses needed to carry the growing foetus.

Other musculoskeletal changes seen in pregnancy include:

exaggerated lordosis of the lower back, forward flexion of the neck and downward movement of the shoulders
joint laxity in the anterior and longitudinal ligaments of the lumbar spine
widening and increased mobility of the sacroiliac joints and pubic symphysis.
